# Distinct antibody and memory B cell responses in SARS-CoV-2 naïve and recovered individuals following mRNA vaccination

**DOI:** 10.1126/sciimmunol.abi6950

**Published:** 2021-04-15

**Authors:** Rishi R. Goel, Sokratis A. Apostolidis, Mark M. Painter, Divij Mathew, Ajinkya Pattekar, Oliva Kuthuru, Sigrid Gouma, Philip Hicks, Wenzhao Meng, Aaron M. Rosenfeld, Sarah Dysinger, Kendall A. Lundgreen, Leticia Kuri-Cervantes, Sharon Adamski, Amanda Hicks, Scott Korte, Derek A. Oldridge, Amy E. Baxter, Josephine R. Giles, Madison E. Weirick, Christopher M. McAllister, Jeanette Dougherty, Sherea Long, Kurt D’Andrea, Jacob T. Hamilton, Michael R. Betts, Eline T. Luning Prak, Paul Bates, Scott E. Hensley, Allison R. Greenplate, E. John Wherry

**Affiliations:** 1 Institute for Immunology, University of Pennsylvania Perelman School of Medicine, Philadelphia, PA, USA.; 2 Immune Health™, University of Pennsylvania Perelman School of Medicine, Philadelphia, PA, USA.; 3 Division of Rheumatology, University of Pennsylvania Perelman School of Medicine, Philadelphia, PA, USA.; 4 Department of Microbiology, University of Pennsylvania Perelman School of Medicine, Philadelphia, PA, USA.; 5 Department of Pathology and Laboratory Medicine, University of Pennsylvania Perelman School of Medicine, Philadelphia, PA, USA.; 6 Department of Systems Pharmacology and Translational Therapeutics, University of Pennsylvania Perelman School of Medicine, Philadelphia, PA, USA.; 7 Parker Institute for Cancer Immunotherapy, University of Pennsylvania Perelman School of Medicine, Philadelphia, PA, USA.

## Abstract

Novel mRNA vaccines for SARS-CoV-2 have been authorized for emergency use. Despite their efficacy in clinical trials, data on mRNA vaccine-induced immune responses are mostly limited to serological analyses. Here, we interrogated antibody and antigen-specific memory B cells over time in 33 SARS-CoV-2 naïve and 11 SARS-CoV-2 recovered subjects. SARS-CoV-2 naïve individuals required both vaccine doses for optimal increases in antibodies, particularly for neutralizing titers against the B.1.351 variant. Memory B cells specific for full-length spike protein and the spike receptor binding domain (RBD) were also efficiently primed by mRNA vaccination and detectable in all SARS-CoV-2 naive subjects after the second vaccine dose, though the memory B cell response declined slightly with age. In SARS-CoV-2 recovered individuals, antibody and memory B cell responses were significantly boosted after the first vaccine dose; however, there was no increase in circulating antibodies, neutralizing titers, or antigen-specific memory B cells after the second dose. This robust boosting after the first vaccine dose strongly correlated with levels of pre-existing memory B cells in recovered individuals, identifying a key role for memory B cells in mounting recall responses to SARS-CoV-2 antigens. Together, our data demonstrated robust serological and cellular priming by mRNA vaccines and revealed distinct responses based on prior SARS-CoV-2 exposure, whereby COVID-19 recovered subjects may only require a single vaccine dose to achieve peak antibody and memory B cell responses. These findings also highlight the utility of defining cellular responses in addition to serologies and may inform SARS-CoV-2 vaccine distribution in a resource-limited setting.

## Teaser

Key differences in both antibody and memory B cell responses to mRNA vaccination were identified based on prior SARS-CoV-2 exposure.

## INTRODUCTION

The COVID-19 pandemic has resulted in hundreds of millions of infections and millions of deaths worldwide (*1*). Novel vaccines have recently been issued emergency use authorization by the FDA and are being widely administered (*2*, *3*). Early data from clinical trials suggest that these vaccines are safe and effective (*4*, *5*); however there is still a paucity of information on how these novel mRNA vaccines elicit immune responses at the cellular and molecular level.

The humoral immune response to infection or vaccination results in two major outcomes: the production of antibodies by antibody secreting cells (ASCs) that can provide rapid serological immunity, and the generation of long-lived memory B cells capable of mounting recall responses (*6*, *7*). If circulating antibodies fail to confer protection to a future exposure, memory B cells drive the recall response by producing new antibodies through forming new ASCs or re-entering germinal centers for additional rounds of somatic hypermutation (*8*, *9*). In the context of acute SARS-CoV-2 infection, immunological memory in the form of antibodies and memory B cells are durable for over 8 months post-symptom onset (*10*–*14*). However, studies on vaccinated individuals have largely focused on measuring binding and/or neutralizing antibodies as primary endpoints (*15*–*17*), and the induction of memory B cells by mRNA vaccines remains poorly understood. Although antibodies are a central component of vaccine efficacy, memory B cells may be important for long-term protection, responses to subsequent infection, and the ability to respond to emerging variant strains (*18*). Furthermore, it is unclear how memory B cell responses relate to serological responses for novel SARS-CoV-2 mRNA vaccines, and how memory B cell responses differ after vaccination in subjects who previously experienced SARS-CoV-2 infection compared to those who are SARS-CoV-2 naïve.

A related question is whether individuals who experienced prior SARS-CoV-2 infection require a second dose of mRNA vaccine. As these individuals have already generated a primary immune response to SARS-CoV-2 during their natural infection, it is possible that a single dose of vaccine could be sufficient to boost antibody and memory B cell responses. This question is particularly relevant in settings of limited vaccine supply and challenging vaccine deployment (*19*). Indeed, several recent studies have indicated that antibody responses can be robustly induced in SARS-CoV-2 experienced individuals, consistent with an anamnestic response (*20*–*23*). Although one study suggests that memory B cells might also be boosted after a single vaccine dose (*24*), it remains unclear how memory B cell responses are affected by the second dose of mRNA vaccine in SARS-CoV-2 naïve versus recovered individuals. These key gaps in our understanding require longitudinal analysis of antibodies together with memory B cell responses after the first and second dose of mRNA vaccine in SARS-CoV-2 naïve and experienced subjects.

Here, we established a longitudinal cohort of SARS-CoV-2 naïve and SARS-CoV-2 recovered individuals who received SARS-CoV-2 mRNA vaccines. From these longitudinal samples, we assessed both circulating antibodies and antigen-specific memory B cells over the course of first and second immunization. We also compared vaccine responses with demographic and clinical metadata, including age and side effects. These data offer new insights into the B cell response to SARS-CoV-2 mRNA vaccines.

## RESULTS

**Fig. 1:**
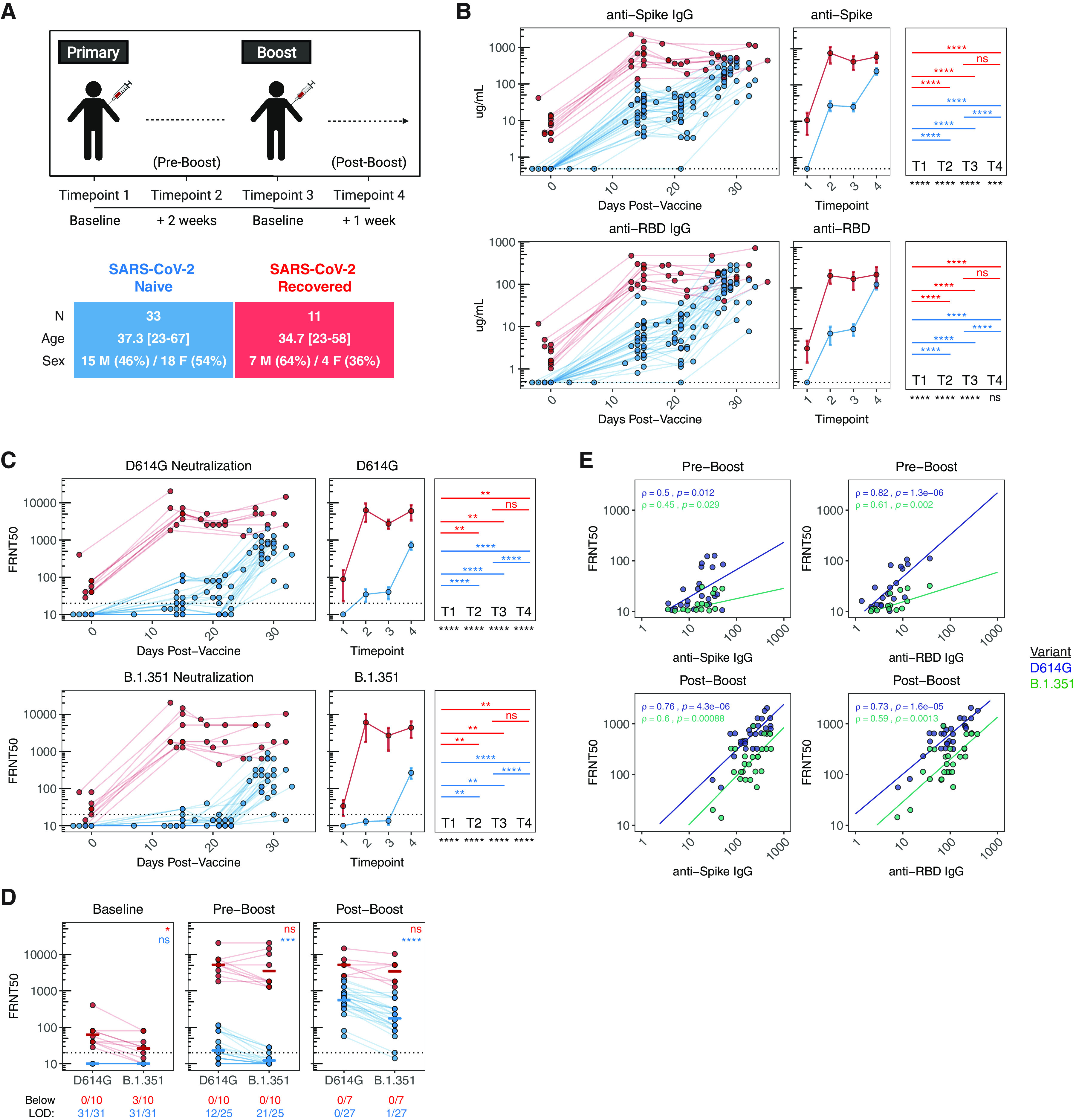
**Antibody responses following mRNA vaccination in SARS-CoV-2 naïve and recovered individuals. A)** UPenn Immune Health COVID vaccine study design. **B)** Concentration of anti-spike and anti-RBD IgG antibodies in vaccinated individuals over time. **C)** Focus reduction neutralization titer 50% (FRNT_50_) of vaccine-induced sera against pseudotyped virus expressing SARS-CoV-2 D614G (wild-type) or B.1.351 (South African) variant spike protein. **D)** Paired analysis of neutralization titers against D614G and B.1.351 in vaccine-induced sera at baseline (timepoint 1), pre-boost (timepoint 2), and post-boost (timepoint 4). **E)** Bivariate analysis of total anti-spike and anti-RBD binding antibodies with pseudovirus neutralization titers against D614G and B.1.351. Associations between total antibody levels and neutralizing ability were calculated using Spearman rank correlation and are shown with linear trend lines. Dotted lines indicate the limit of detection (LOD) for the assay. Statistics were calculated using unpaired Wilcoxon test (comparisons between timepoints and comparisons between naïve and recovered) or paired Wilcoxon test (comparisons between D614G and B.1.351) with Holm correction for multiple comparisons. Blue and red values indicate statistical comparisons within naïve or recovered groups. Black values indicate statistical comparisons between naïve or recovered groups.

For this study, we recruited 44 healthy individuals (i.e., no self-reported chronic health conditions) who received SARS-CoV-2 mRNA vaccines (Pfizer BNT162b2 or Moderna mRNA-1273) at the University of Pennsylvania Health System. Full cohort information is described in **figure S1**. Of this cohort, 11 individuals had a prior SARS-CoV-2 infection, ranging from 65 to 275 days prior to vaccination. Peripheral blood samples were collected for immunological analysis at 4 key timepoints (**Fig. 1A**): pre-vaccine baseline (timepoint 1), 2 weeks following the first dose (timepoint 2), the day of second dose (timepoint 3), and 1 week following the second dose (timepoint 4). This study design allowed us to investigate the kinetics of immune responses following both primary and secondary immunizations.

### Antibody responses to SARS-CoV-2 mRNA vaccination

We first measured circulating antibody responses in longitudinal serum samples by ELISA. At baseline, SARS-CoV-2 naïve individuals had undetectable levels of IgG antibodies specific for either full-length spike protein or the spike receptor binding domain (RBD) (**Fig. 1B**). Primary vaccination induced a significant increase in SARS-CoV-2-specific antibodies, that was further enhanced by the booster dose (**Fig. 1B**). In contrast, all SARS-CoV-2 recovered individuals had detectable levels of anti-spike and anti-RBD IgG at baseline, and these antibody responses were significantly increased after the first dose of vaccine (**Fig. 1B**). However, in SARS-CoV-2 recovered subjects, there was no additional increase in antibody levels following the second vaccine dose (**Fig. 1B**). Notably, the levels of anti-RBD IgG were similar in the SARS-CoV-2 naïve and SARS-CoV-2 recovered individuals at 1 week post-boost (timepoint 4) (**Fig. 1B**).

In addition to total spike- and RBD-binding antibody, we further assessed antibody function using a pseudovirus neutralization assay. Specifically, we tested the ability of vaccine-induced sera to neutralize pseudotyped virus expressing either the D614G (the initial dominant strain at the time of the study) spike protein or the B.1.351 variant (commonly referred to as the South African variant) spike protein. SARS-CoV-2 naïve individuals had a moderate response to primary immunization with ~50% of participants developing detectable levels of neutralizing antibodies against D614G two weeks post-primary (**Fig. 1C-D**). In contrast, primary immunization was largely ineffective to induce functional antibodies against the B.1.351 variant with only 4/25 individuals developing neutralizing titers above limit of detection over the same time frame (**Fig. 1C-D**). Neutralizing titers were significantly increased after the second dose in SARS-CoV-2 naïve individuals, with all participants achieving neutralization against D614G and 26/27 achieving detectable neutralization against B.1.351 at 7 days post-boost (**Fig. 1C-D**). Consistent with anti-spike and anti-RBD antibody levels, SARS-CoV-2 experienced individuals had a robust increase in neutralizing antibodies following primary immunization, with no further increase in neutralization titers against D614G and B.1.351 after the second dose (**Fig. 1C**). Interestingly, the first dose of vaccine also appeared to resolve baseline differences in neutralization between D614G and B.1.351 in this group (**Fig. 1D**).

Based on these data, we quantified the relationship between total antibody levels and neutralization ability in SARS-CoV-2 naïve individuals to assess the relative quality of antibodies induced by the first and second dose of mRNA vaccine. Before the second dose, anti-spike antibodies were only moderately correlated with neutralizing titers against D614G, with further dropoff for the B.1.351 variant (**Fig. 1E**). Pre-boost anti-RBD antibodies were more predictive of neutralization titers against D614G and B.1.351 (**Fig. 1E**) than anti-spike antibodies. Both anti-spike and anti-RBD antibodies correlated more strongly with neutralizing titers against D614G and B.1.351 after the second dose (**Fig. 1E**), indicating a marked improvement in the quality of the antibody response. Together, these data supported the importance of a 2 dose-regimen for effective antibody responses, especially against the B.1.351 variant, in SARS-CoV-2 naïve individuals. Conversely, a single dose of vaccine was able to achieve highly effective antibody responses in SARS-CoV-2 recovered individuals with no further improvement post-boost.

### Memory B Cell responses to SARS-CoV-2 mRNA vaccination

We next asked how mRNA vaccination impacted the responses of memory B cells specific for SARS-CoV-2. To address this question, we developed a flow cytometric assay using a combination of fluorescently labeled antigens as probes to track the induction of virus-specific memory B cells in longitudinal PBMC samples (**figure S2A**) (*11*, *13*, *25*). Analysis of bulk B cell populations revealed no change in the frequency of naïve, non-naïve, or memory B cells across the timecourse of vaccination, or between SARS-CoV-2 naïve and recovered individuals (**figure S2B**), highlighting the stability of the overall B cell compartment.

**Fig. 2:**
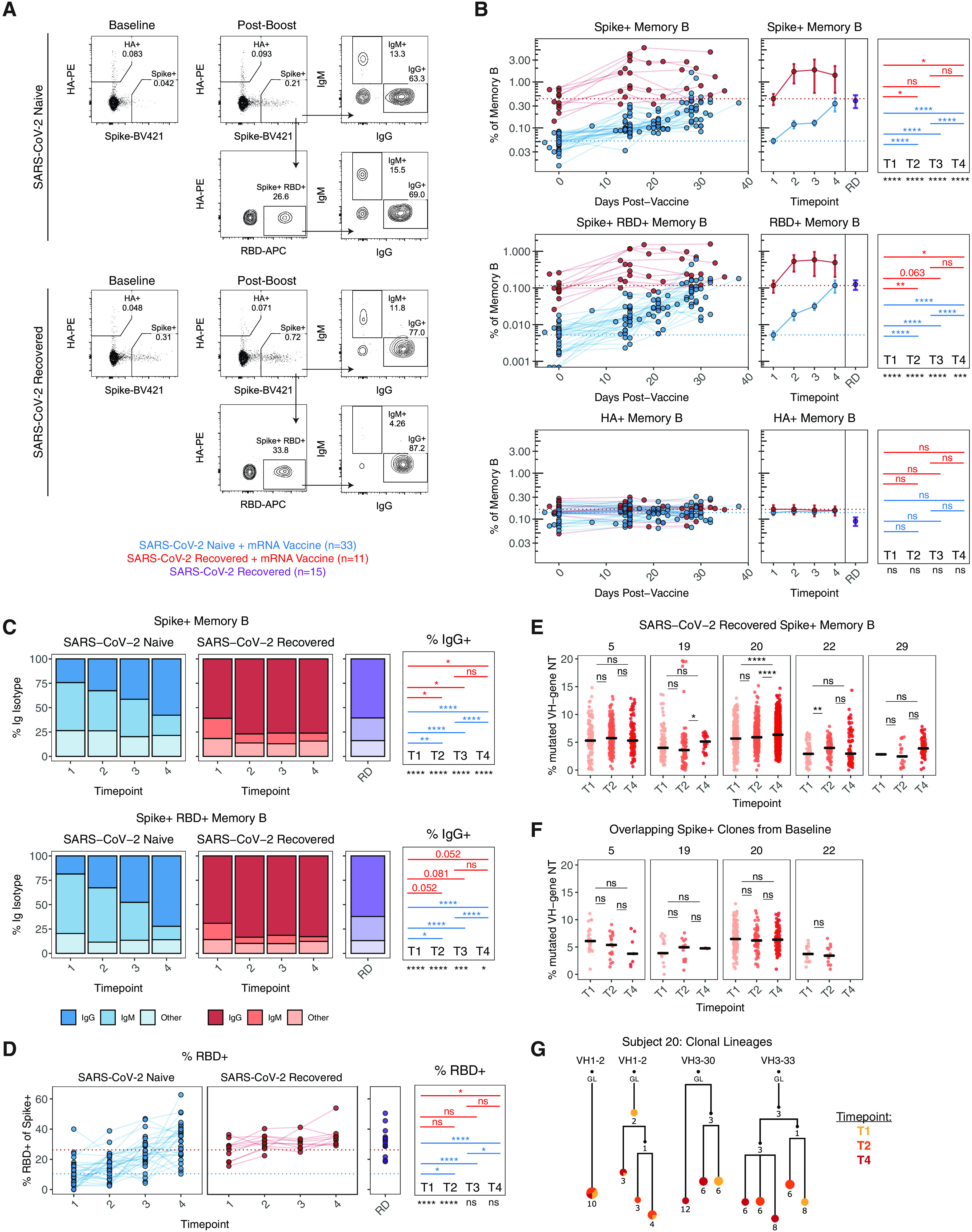
**Antigen-specific memory B cell responses following mRNA vaccination in SARS-CoV-2 naïve and recovered individuals. A)** Gating strategy and representative plots for flow cytometric analysis of SARS-CoV-2-specific B cells. **B)** Frequency of spike^+^, spike^+^/RBD^+^, and HA^+^ memory B cells over time in vaccinated individuals. Data are represented as frequency of antigen-specific cells in the total memory B cell compartment. **C)** Frequency of IgG and IgM isotypes over time in the antigen-specific memory B cell compartments. **D)** Frequency of RBD^+^ memory B cells over time in vaccinated individuals, as a percentage of spike^+^ memory B cells. **E)** Somatic hypermutation (SHM) status of spike+ memory B cell clones over time in SARS-CoV-2 recovered individuals. Data are represented as percent of VH-gene nucleotides that are mutated. **F)** SHM of productive spike-binding clones sampled at timepoint 1 which were also found in at least one other timepoint. Clones with fewer than 10 copies in each patient were excluded. **G)** Clonal evolution of spike-binding memory B cell lineages that were present prior to vaccination in a recovered individual. For representative lineages, numbers refer to mutations compared to the preceding vertical node. Colors indicate timepoint, black dots indicate inferred nodes, and size is proportional to sequence copy number; GL = germline sequence. All panels: Dotted lines indicate the mean at baseline. RD = non-vaccinated, SARS-CoV-2 recovered donors. Statistics were calculated using unpaired Wilcoxon test (comparisons between timepoints and comparisons between naïve and recovered) with Holm correction for multiple comparisons. Blue and red values indicate statistical comparisons within naïve or recovered groups. Black values indicate statistical comparisons between naïve or recovered groups.

Despite a stable frequency of total memory B cells, there were marked changes in SARS-CoV-2 antigen-specific B cell populations in response to vaccination. Consistent with the antibody data, SARS-CoV-2 naïve individuals had minimal spike-specific memory B cells at baseline, whereas SARS-CoV-2 recovered individuals had a significant population of spike-specific memory B cells ranging from ~0.15-0.8% of total memory B cells (**Fig. 2A-B**). Memory B cells targeting the spike RBD followed a similar trend and the frequency of these antigen-specific memory B cells was comparable to a separate cohort of non-vaccinated SARS-CoV-2 recovered donors (**Fig. 2A-B**). After primary immunization, SARS-CoV-2 naïve individuals had a significant increase in spike-specific and RBD-specific memory B cells over baseline (**Fig. 2B**). These memory B cells were also significantly boosted after adminstration of the second vaccine dose, approaching the levels of memory B cells observed in non-vaccinated SARS-CoV-2 recovered donors (**Fig. 2B**). In contrast, SARS-CoV-2 recovered individuals had a robust expansion of spike- and RBD-specific memory B cells following primary immunization, but had no additional boosting after the second vaccine dose (**Fig. 2B**). As a control we also examined the frequency of influenza hemagglutinin (HA)-specific memory B cells in both SARS-CoV-2 naïve and recovered individuals following SARS-CoV-2 vaccination. The frequency of these antigen-unrelated memory B cells remained stable throughout the mRNA vaccination timecourse (**Fig. 2B**), confirming the specificity of this memory B cell assay. Together, these results demonstrated robust induction of SARS-CoV-2-specific memory B cells by two doses of mRNA vaccine in SARS-CoV-2 naïve subjects. In contrast, a single dose of mRNA vaccine amplified pre-existing antigen-specific memory B cells in SARS-CoV-2 recovered subjects, with no additional quantitative benefit after the second vaccine dose.

We further analyzed the phenotype of SARS-CoV-2 specific memory B cells. On day 15 after primary immunization, ~25-30% of spike-specific memory B cells were IgG^+^ and ~40-50% were IgM^+^ in SARS-CoV-2 naïve individuals (**Fig. 2C**). The frequency of IgG^+^ memory B cells increased to >50% following the second dose of vaccine in these subjects (**Fig. 2C**), consistent with a qualitative improvement in memory B cells after the boost. Conversely, in SARS-CoV-2 recovered individuals, ~60-70% of spike-specific memory B cells detected prior to vaccination were IgG^+^ (**Fig. 2C**). Although the frequency of IgG^+^ memory B cells increased slightly to ~75% following the first dose of vaccine, further increases were not observed after the second immunization (**Fig. 2C-D**). A similar pattern of IgG frequency was observed for RBD-specific memory B cells (**Fig. 2C**). In addition, the fraction of spike-specific memory B cells that recognized RBD remained stable over time in SARS-CoV-2 recovered individuals. In SARS-CoV-2 naïve subjects, the fraction of the overall spike-specific memory B cell response that was focused on RBD increased over time, becoming equivalent to that observed in SARS-CoV-2 recovered individuals after the second vaccine dose (**Fig. 2D**). Overall, these data indicated a qualitative benefit to the virus-specific memory B cell response following both doses of vaccine in SARS-CoV-2 naïve individuals, and qualitative improvement following the first but not the second vaccine dose in SARS-CoV-2 recovered subjects.

Finally, we sorted spike^+^ memory B cells from 5 recovered donors at baseline (timepoint 1), post-primary (timepoint 2), and post-boost (timepoint 4) for B cell receptor (BCR) sequencing to further evaluate potential changes in the memory B cell response induced by vaccination. Somatic hypermutation (SHM) is a process of DNA point hypermutation that occurs in immunoglobulin variable gene sequences and usually accompanies T cell-dependent B cell responses within germinal centers (*26*). Accordingly, SHM is a frequently used marker for the evaluation of immune memory (*27*). Here, SHM was calculated as the average percentage of mutated VH-gene nucleotides in each clone, counting each clone only once. Full sequencing information, including the number of clones identified for each sample, is listed in **table S3**. Mutational analysis of total spike-binding memory clones revealed a modest shift toward higher SHM at the post-primary and post-boost timepoint in some individuals (**Fig. 2E**); however, there was no clear pattern across the 5 individuals measured. To determine if SHM changed within pre-existing spike-binding clones, we next looked for high-copy spike-binding clones that were shared between the baseline timepoint and at least one other timepoint. These clones, which were present before the first vaccine dose, presumably arose during the initial infection with SARS-CoV-2. Subject 29 was not included in this analysis because there was only one clone that met the copy number cutoff. SHM levels in the overlapping clones did not increase after vaccination (**Fig. 2F**). The stability of SHM could also be seen within lineage trees for subject 20, who had the largest number of clones sampled. Specifically, the nodes (sequence variants) within lineages exhibited mixing between the timepoints, and where they were separate, they were not consistently found at higher frequencies in parts of the trees with higher levels of SHM (**Fig. 2G****, figure S3**). These data suggested that pre-existing spike-specific memory clones in SARS-CoV-2 recovered individuals did not increase their level of SHM in response to either dose of vaccine.

### Demographic and clinical factors associate with B cell responses to SARS-CoV-2 mRNA vaccination

**Fig. 3:**
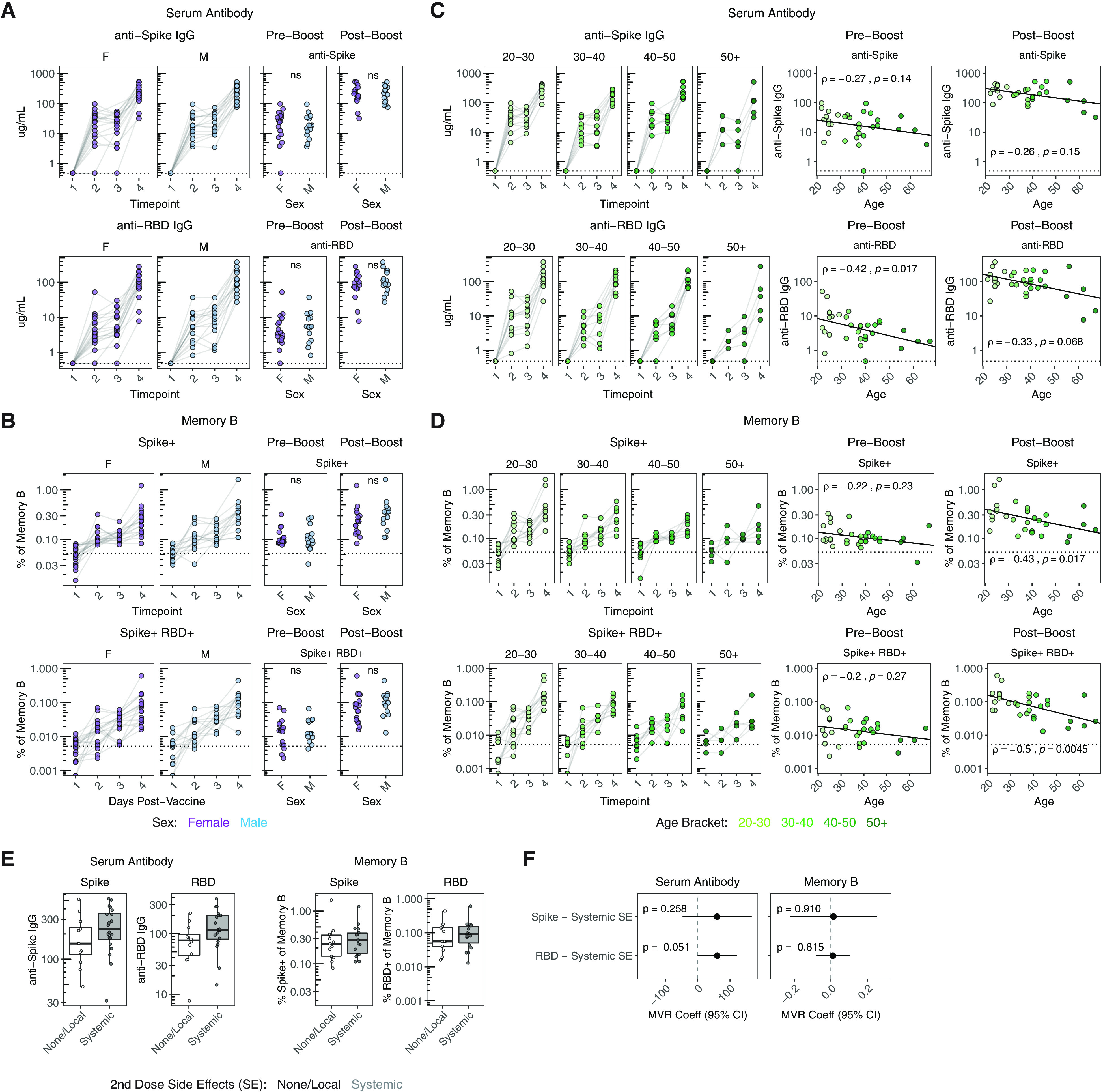
**Association of age and side-effects with B cell responses following mRNA vaccination. A, C)** Concentration of anti-spike and anti-RBD IgG antibodies over time compared with sex (A) and age (C) in SARS-CoV-2 naïve individuals. Dotted lines indicate the limit of detection for the assay. **B, D)** Frequency of spike^+^ and spike^+^/RBD^+^ memory B cells over time compared with sex (B) and age (D) in SARS-CoV-2 naïve individuals. Dotted lines indicate the mean frequency of cells at baseline. Pre-boost indicates samples collected at timepoint 2 (~15 days post-primary vaccination). Post-boost indicates samples collected at timepoint 4 (~7 days post-secondary vaccination). Statistics for sex were calculated using Wilcoxon test. Associations with age were calculated using Spearman rank correlation and are shown with linear trend lines. **E)** Concentration of anti-spike and anti-RBD IgG antibodies and frequency of spike^+^ and spike^+^/RBD^+^ memory B cells at the post-boost timepoint compared with self-reported side effects after the second dose. Reactogenicity was categorized into either no/local symptoms or systemic symptoms. **F)** Multivariable linear regression between antibody or memory B cell responses and side effects, controlling for sex and age. Data are represented as estimated regression coefficients with a 95% confidence interval.

In addition to prior SARS-CoV-2 exposure, we also investigated associations between other demographic and clinical metadata with vaccine-induced B cell responses. Several previous studies have reported a negative association between age and vaccine-induced antibody titers after a single dose of mRNA vaccines (*28*, *29*). We therefore investigated potential relationships between sex or age and B cell responses after one or two doses of vaccine. In our cohort of SARS-CoV-2 naïve vaccinees, there were no associations between sex and antibody or memory B cell responses (**Fig. 3A****, ****3B**). While there was no association between age and anti-spike IgG after the first immunization (i.e., pre-boost), there was a trend toward a negative relationship between age and pre-boost RBD-specific IgG (**Fig. 3C**). Antibody for both spike and RBD had a similarly negative, but statistically insignificant, correlation with age after the second vaccine dose (**Fig. 3C**). However, there was a clear negative correlation between the post-boost frequency of antigen-specific memory B cells and age (**Fig. 3D**). Although this relationship represented weaker induction of memory B cells with older age, all age groups still displayed an increase in the frequency of SARS-CoV-2 specific memory B cells compared to pre-vaccine baseline (**Fig. 3C-D**). There was also no change in the frequency of total memory B cells by sex or age, indicating the antigen-specific nature of this effect (**figure S4**). Although our cohort is not extensively enriched in those over 50 years old and does not directly address elderly vaccinees, these data pointed to potentially relevant age-related changes in immune response to vaccination.

An additional question is whether vaccine-induced side effects have any relationship to immune responses (*20*). We addressed this question by comparing vaccine-induced antibody and memory B cell responses in subjects with or without self-reported systemic side effects (i.e., fever, chills, headache, fatigue, myalgia; **figure S1C**). In SARS-CoV-2 naïve vaccinees with systemic side-effects following the second dose, there was a trend toward an increase in antibody responses at the post-boost timepoint (**Fig. 3E**). Such a trend was not observed for the memory B cell response (**Fig. 3E**). We further investigated the potential association between reactogenicity and increased antibody response using a multivariate regression to control for the effects of sex and age. This multivariate analysis similarly revealed a positive association of systemic side effects with anti-spike and anti-RBD antibody levels 7 days after the booster immunization (**Fig. 3F**). Although these data only represent a statistical trend (p=0.051), they do provoke questions about potential relationships between early vaccine-induced inflammation and the induction of antibody responses that should be addressed in future studies.

### Relationships between antibody and memory B cell responses to SARS-CoV-2 mRNA vaccination

**Fig. 4:**
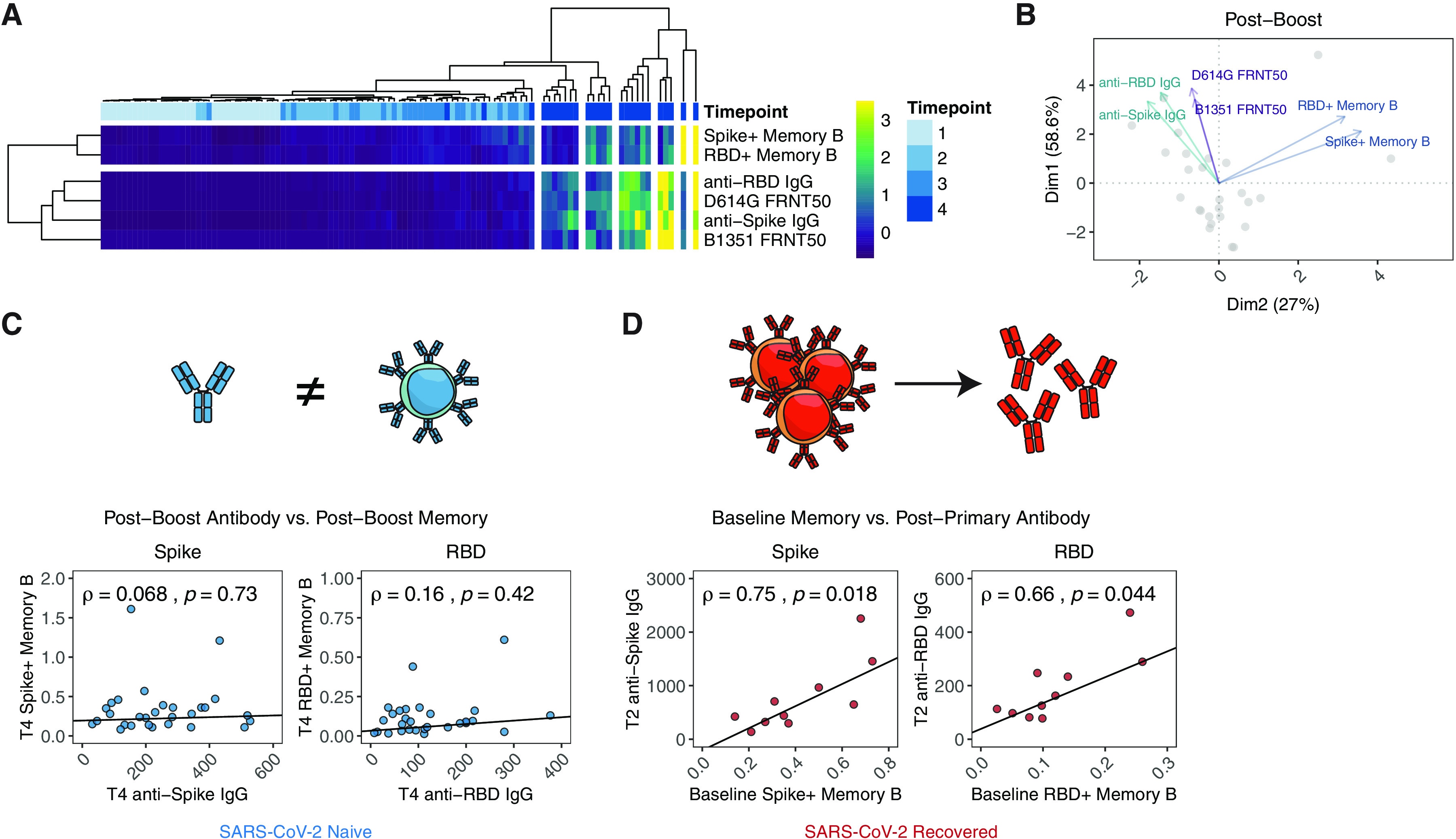
**Antigen-specific memory B cells were a distinct measure of vaccine efficacy and correlated to antibody recall responses. A)** Heatmap and hierarchical clustering of vaccine-induced antibody and memory B cell responses. **B)** Principal component analysis and biplot of vaccine-induced antibody and memory B cell responses. **C)** Association of post-boost (timepoint 4) antibody levels with post-boost (timepoint 4) antigen-specific memory B cell frequencies in SARS-CoV-2 naïve individuals. **D)** Association of baseline (timepoint 1) antigen-specific memory B cell frequencies with post-primary vaccination (timepoint 2) antibody levels in SARS-CoV-2 recovered individuals. Illustrations in (C) and (D) represent the corresponding immune relationship. Associations between immunological parameters were calculated using Spearman rank correlation and are shown with non-parametric trend lines (Theil-Sen estimator).

Finally, we investigated the potential relationships between antibody and memory B cell responses. To address this question, we first performed hierarchical clustering of vaccine-induced B cell responses in SARS-CoV-2 naïve subjects. As expected, post-boost (timepoint 4) samples clustered away from the earlier timepoints, with some sub-grouping of patients based on the relative magnitude of antibody and memory B cell responses (**Fig. 4A**). Hierarchical clustering of the different readouts of antigen-specific humoral immunity also revealed that antibodies and memory B cells clustered separately (**Fig. 4A**). We next performed a principal component analysis (PCA) of post-boost B cell responses. Antibody and memory B cell measurements had distinct contributions to the first 2 principal components, with total binding antibodies and neutralizing titers primarily contributing to dimension 1 (Dim1) and memory cells primarily contributing to dimension 2 (Dim2) (**Fig. 4B**). Based on these data, we further examined the relationship between circulating antibody responses and corresponding memory B cell responses after two doses of vaccine in a bivariate analysis. Despite strong induction of both spike- and RBD-specific antibody and memory B cells in these subjects, there was no association between the levels of post-boost antibodies and B cell memory (**Fig. 4C**), indicating that short-term serological responses and memory B cell responses may be distinct immunological features of response to mRNA vaccination. Similarly, pre-vaccine baseline antibody levels did not correlate with baseline memory B cell frequencies in SARS-CoV-2 recovered individuals (**figure S5A**). We next asked which measure of humoral immunity predicted antibody recall responses post-vaccination. Interestingly, the baseline levels of SARS-CoV-2-specific antibody correlated with the level of anti-spike, but not anti-RBD antibody achieved after primary vaccine in SARS-CoV-2 recovered donors (**figure S5B**). However, the baseline frequency of antigen-specific memory B cells strongly correlated with post-primary vaccination antibody levels for both spike and RBD (**Fig. 4D**), consistent with the notion that these pre-vaccination memory B cells are major contributors to the SARS-CoV-2 antibody recall response. These data highlight the importance of measuring antigen-specific memory B cells in addition to serologic antibody evaluation as an immunological correlate of vaccine-induced immunity.

**Fig. 5:**
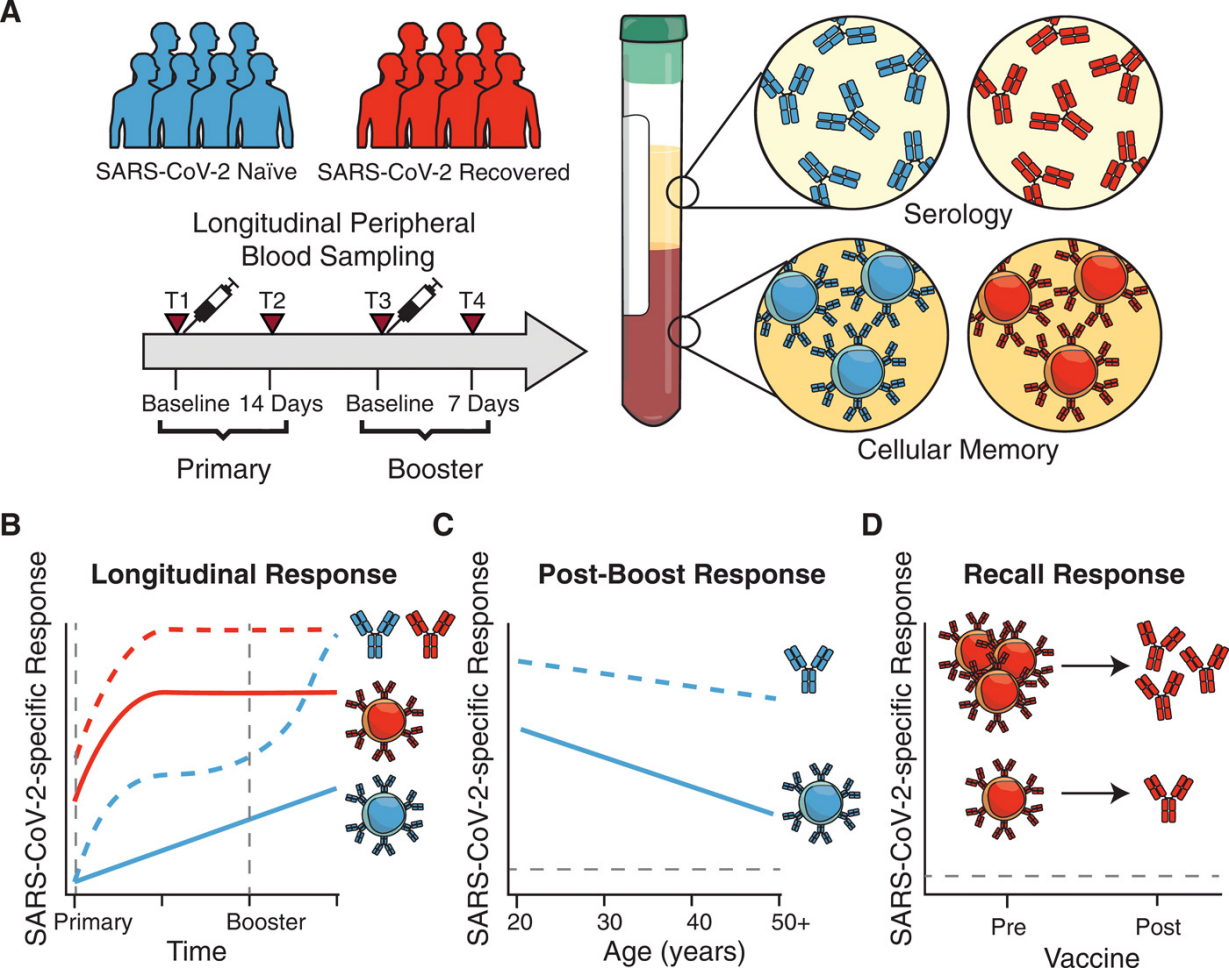
**Study summary and key findings**). **A)** Cohort design and objectives. Longitudinal samples were collected from SARS-CoV-2 naïve and recovered individuals and measured for both antibodies and memory B cells. **B)** Distinct patterns of antibody and memory B cell responses to mRNA vaccination in SARS-CoV-2 naïve and recovered individuals. **C)** Age-associated differences in antibody and memory B cell responses to mRNA vaccination. **D)** Baseline memory B cells in SARS-CoV-2 recovered individuals contribute to recall responses following mRNA vaccination.

Overall, we tracked antibody and antigen-specific memory B cells over time following SARS-CoV-2 mRNA vaccination and documented robust priming of antibody as well as memory B cell responses (**Fig. 5A**). Our analysis revealed key differences in vaccine-induced immune response between SARS-CoV-2 naïve and recovered subjects after the first versus second dose of vaccine. (**Fig. 5B**). SARS-CoV-2 naive individuals required two doses of vaccine to achieve optimal priming of antibodies, including neutralizing antibodies to the B.1.351 strain and memory B cells. In contrast, SARS-CoV-2 recovered subjects may only require a single vaccine dose to achieve peak antibody and memory B cell responses. We also revealed age-related differences in vaccine-induction of immune responses (**Fig. 5C**) and highlighted the importance of memory B cells in mounting recall antibodies in SARS-CoV-2 recovered subjects (**Fig. 5D**).

## DISCUSSION

Here, we demonstrated that mRNA vaccines to SARS-CoV-2 induced robust antibody and memory B cell responses to full-length spike and the RBD. These results are encouraging for both short- and long-term vaccine efficacy and add to our understanding of SARS-CoV-2 mRNA vaccine-induced immune responses in several ways. First, our serological data are consistent with several other recent studies (*20*, *21*, *23*, *24*, *28*, *29*) indicating robust boosting of antibody responses in SARS-CoV-2 recovered subjects after the first vaccine dose, but little benefit to antibody levels after the second vaccine dose. This finding was also reflected in the observation that neutralizing titers against both D614G and the B.1.351 South African variant reached a peak after the first dose in recovered subjects. Moreover, we found a similar effect for virus-specific memory B cells, identifying a quantitative and qualitative plateau in vaccine-induced memory B cells in COVID-19 recovered subjects after the first dose of vaccine with little additional change to the memory B cell response following booster vaccination. These data suggest that only a single vaccine dose in individuals confirmed to have previously been infected with SARS-CoV-2 may be enough to induce antibody and memory B cell responses.

The data presented document key differences in immune responses associated with vaccine efficacy in SARS-CoV-2 naïve versus SARS-CoV-2 recovered individuals. However, with a study of this size designed for deep immunological analysis, it was not possible to directly address protection or true vaccine efficacy. Accordingly, larger-scale clinical studies would be necessary to fully examine the question of a one or two dose regimen in SARS-CoV-2 recovered individuals. Our cohort also consisted of individuals who were not hospitalized during their SARS-CoV-2 infections, and it may be necessary to address this question of one versus two doses of vaccine in individuals who experienced more severe COVID-19. Moreover, there may be practical challenges to identifying SARS-CoV-2 recovered individuals based on self-reported infection or laboratory confirmed tests such as RT-PCR or serology. Despite these limitations, the robust boosting of both antibody and memory B cells in these subjects after one dose may have implications for vaccine distribution in settings where supply is limited.

An additional question is whether the second vacccine dose in recovered individuals has other immunological effects not reflected in overall antibody titers or memory B cell frequency and phenotype. Given the relatively short timeframe of this study, future studies will be necessary to evalute durability of immune responses in these subjects and investigate potential differences in long-term immunological memory. Our data indicates that pre-formed spike-binding memory B cell clones that were resampled at multiple time points did not have obvious increases in SHM, suggesting that the B cell clones boosted by mRNA vaccination in SARS-CoV-2 recovered individuals have stable SHM profiles. However, these analyses were only performed on a small number of individuals and samples were limited to only the first few weeks following vaccination. Thus, it will be important to determine if these clones evolve and undergo further SHM over time as occurs after natural SARS-CoV-2 infection (*9*, *30*, *31*). Even small changes in SHM may be biologically relevant, as somatically mutated clones can exhibit higher degrees of cross-protection against different mutant strains of the virus (*30*). It is also possible that other post-germinal center clones emerge later in the memory phase. Lastly, it is possible that booster vaccination has some beneficial effects on virus-specific T cell responses in SARS-CoV-2 recovered individuals. Given the capacity of mRNA vaccines to induce CD4+ T cell responses (*32*), this topic merits further investigation.

In contrast to SARS-CoV-2 recovered subjects, SARS-CoV-2 naïve individuals demonstrated considerable benefit to antibody and memory B cell responses from the second dose of mRNA vaccine. It is possible that some of this benefit would occur over time in the absence of a booster vaccination; however, the spike- and RBD-specific antibody levels appeared to plateau between the first and second doses of vaccine before increasing again following booster vaccination. Additionally, only half of SARS-CoV-2 naïve individuals had neutralizing antibodies to wild type virus, and only 2/25 had neutralizing antibodies to the B.1.351 variant after the first dose of vaccine, whereas nearly all subjects achieved neutralizing antibodies following the boost. Moreover, the frequency of memory B cells that were IgG^+^ and the fraction that was focused on RBD both increased after booster vaccination, indicating an improvement in the quality of the memory B cell response. Together, these data are consistent with the need for a two dose mRNA vaccine schedule in SARS-CoV-2 naïve individuals to achieve optimal levels of humoral immunity, including neutralizing antibodies against the B.1.351 variant.

We also observed a negative association of age with induction of B cell memory. Others have reported a negative association between age and serum antibody titers after a single mRNA vaccine dose (*28*, *29*). We found a similar trend for antibodies following two doses of mRNA vaccination, but this did not reach statistical significance for our cohort. However, the magnitude of the memory B cell response following the second dose was lower with increased age, confirming age as a key variable in mRNA vaccine-induced immunity. It remains unclear if the age-associated effect on memory B cell induction represents a true difference in the magnitude of response or a difference in kinetics that will resolve at later timepoints. It is also challenging to define an exact threshold for how much immunological memory is sufficient to provide long-term protection. Although all subjects, regardless of age, had significant humoral and memory B cell responses to vaccination, these data highlight a need to further understand the age-related changes in responses to mRNA vaccination (*33*). In examining correlates of vaccine-induced immune responses, we also uncovered a trend suggesting that vaccine-induced side effects may be related to post-vaccination serum antibodies, but not memory B cells. Although more data are needed, it is possible that systemic inflammation early after vaccination could contribute to an initial induction of antibody with less of an impact on the development of memory B cells. Larger cohorts and more quantitative measures of vaccine-induced side effects may further clarify these relationships.

Finally, these analyses highlight the importance of interrogating vaccine-induced memory B cell responses alongside serological analyses. Specifically, we found no relationship between post-vaccination serum antibody levels and memory B cells in SARS-CoV-2 naïve subjects, indicating that antibody and memory B cell induction may be independent features of the immune response to mRNA vaccination. Previous work has found that antibodies and memory B cells correlate for some vaccines or antigens, but do not correlate for many others (*34*). Current research on SARS-CoV-2 vaccines has largely focused on measuring circulating antibodies without measuring memory B cells, which are important for durability of immune memory and potential recall responses to infection or future booster . Indeed, pre-existing memory B cells in SARS-CoV-2 recovered subjects correlated strongly with post-vaccination antibody levels in our cohort, underscoring the immunological connection between memory B cells and antibody recall responses (*35*). Taken together, our findings highlight the importance of evaluating memory B cells in addition to serologies to more completely characterize humoral immunity. Although high circulating titers of neutralizing antibodies are common surrogates of protective immunity, there are many scenarios where circulating antibodies may not achieve sterilizing immunity and additional immune responses from memory cells will be necessary (*36*). For example, high dose viral innoculums may require rapid generation of additional antibody from memory B cells. Moreover, if circulating antibodies wane over time, our data suggest that durable memory B cells are likely to provide a rapid source of protective antibody upon SARS-CoV-2 re-exposure. Lastly, infection with variant strains that partially escape neutralization by existing circulating antibodies (*37*–*39*) might require strong memory B cell populations that can re-seed germinal centers and diversify to respond to novel spike antigens (*40*).

In summary, our analysis of antibodies and cellular memory reveals distinct responses to SARS-CoV-2 mRNA vaccines based on prior history of infection. The addition of memory B cells in this analysis, both in terms of frequency and phenotype, provides complemenary data that strengthens current serology-based evidence (*20*, *21*, *23*, *24*, *28*, *29*) for a single-dose vaccine schedule in COVID-19 recovered individuals. We also find associations of vacccine-induced immune responses with age and side effects, which may have relevance for future booster vaccines and public health campaigns. Thus, our study provides insight into the underlying immunobiology of mRNA vaccines in humans and may have implications for vaccination strategies in the future.

## MATERIALS AND METHODS

### Study Design

The objective of this study was to define antigen-specific measures of humoral immunity in peripheral blood of healthy adults following SARS-CoV-2 mRNA vaccination. A secondary objective of this study was to compare antigen-specific responses to mRNA vaccination in SARS-CoV-2 naïve and recovered individuals. This study began in December 2020 and is continuing to enroll participants.

### Recruitment and Clinical Sample Collection

44 individuals (33 SARS-CoV-2 naïve, 11 SARS-CoV-2 recovered) were consented and enrolled in the study with approval from the University of Pennsylvania Institutional Review Board (IRB# 844642). All participants were otherwise healthy and based on self-reported health screening did not report any history of chronic health conditions. Subjects were stratified based on self-reported and laboratory evidence of a prior SARS-CoV-2 infection. Of the self-reported naïve subjects, one individual was found to have positive SARS-CoV-2 specific antibodies and memory B cells at baseline and was retroactively classified as SARS-CoV-2 recovered. All subjects received either Pfizer (BNT162b2) or Moderna (mRNA-1273) mRNA vaccines. Samples were collected at 4 timepoints: baseline, 2 weeks post-primary immunization, day of booster immunization, and 1 week post-booster immunization. Timepoints were chosen a priori to capture the peak antigen-specific response for primary (*41*) and secondary responses (*42*, *43*) in SARS-CoV-2 naïve individuals. 80-100mL of peripheral blood samples and clinical questionnaire data were collected at each study visit. Full cohort and demographic information is provided in **figure S1**. Non-vaccinated recovered COVID-19 donors (RD) were adults with a prior positive COVID-19 PCR test by self-report who met the definition of recovery by the Centers for Disease Control (*44*).

### Sample Processing

Venous blood was collected into sodium heparin and EDTA tubes by standard phlebotomy. Blood tubes were centrifuged at 3000rpm for 15 min to separate plasma. Heparin and EDTA plasma were stored at -80°C for downstream antibody analysis. Remaining whole blood was diluted 1:1 with RPMI + 1% FBS + 2mM L-Glutamine + 100 U Penicillin/Streptomycin and layered onto SEPMATE tubes (STEMCELL Technologies) containing lymphoprep gradient (STEMCELL Technologies). SEPMATE tubes were centrifuged at 1200 g for 10 min and the PBMC fraction was collected into new tubes. PBMCs were then washed with RPMI + 1% FBS + 2mM L-Glutamine + 100 U Penicillin/Streptomycin and treated with ACK lysis buffer (Thermo Fisher) for 5 min. Samples were washed again with RPMI + 1% FBS + 2mM L-Glutamine + 100 U Penicillin/Streptomycin, filtered with a 70μm filter, and counted using a Countess automated cell counter (Thermo Fisher). Aliquots containing 5x10^6^ PBMCs were cryopreserved in fresh 90% FBS 10% DMSO.

### Detection of SARS-CoV-2-Specific Antibodies

Plasma samples were tested for SARS-CoV-2-specific antibody by enzyme-linked immunosorbent assay (ELISA) as previously described (*45*). The estimated sensitivity of the test is 100% [95% confidence interval (CI), 89.1 to 100.0%], and the specificity is 98.9% (95% CI, 98.0 to 99.5%) (*45*). Plasmids encoding the recombinant full-length spike protein and the RBD were provided by F. Krammer (Mt. Sinai) and purified by nickel-nitrilotriacetic acid resin (Qiagen). ELISA plates (Immulon 4 HBX, Thermo Fisher Scientific) were coated with PBS or 2 ug/mL recombinant protein and stored overnight at 4C. The next day, plates were washed with phosphate-buffered saline containing 0.1% Tween-20 (PBS-T) and blocked for 1 hour with PBS-T supplemented with 3% non-fat milk powder. Samples were heat-inactivated for 1 hour at 56C and diluted in PBS-T supplemented with 1% non-fat milk powder. After washing the plates with PBS-T, 50 uL diluted sample was added to each well. Plates were incubated for 2 hours and washed with PBS-T. Next, 50 uL of 1:5000 diluted goat anti-human IgG-HRP (Jackson ImmunoResearch Laboratories) or 1:1000 diluted goat anti-human IgM-HRP (SouthernBiotech) was added to each well and plates were incubated for 1 hour. Plates were washed with PBS-T before 50 uL SureBlue 3,3′,5,5′-tetramethylbenzidine substrate (KPL) was added to each well. After 5 min incubation, 25 uL of 250 mM hydrochloric acid was added to each well to stop the reaction. Plates were read with the SpectraMax 190 microplate reader (Molecular Devices) at an optical density (OD) of 450 nm. Monoclonal antibody CR3022 was included on each plate to convert OD values into relative antibody concentrations. Plasmids to express CR3022 were provided by I. Wilson (Scripps).

### SARS-CoV-2 Neutralization Assay

*Production of VSV pseudotypes with SARS-CoV-2 Spike:* 293T cells plated 24 hours previously at 5 X 10^6^ cells per 10 cm dish were transfected using calcium phosphate with 35 μg of pCG1 SARS-CoV-2 S D614G delta18 or pCG1 SARS-CoV-2 S B.1.351 delta 18 expression plasmid encoding a codon optimized SARS-CoV-2 S gene with an 18 residue truncation in the cytoplasmic tail (kindly provided by Stefan Pohlmann). Twelve hours post transfection the cells were fed with fresh media containing 1mM sodium butyrate to increase expression of the transfected DNA. 24 hours after transfection, the SARS-CoV-2 spike expressing cells were infected for two hours with VSV-G pseudotyped VSVΔG-RFP at an MOI of ~1. Virus containing media was removed and the cells were re-fed with media without serum. Media containing the VSVΔG-RFP SARS-CoV-2 pseudotypes was harvested 28-30 hours after infection and clarified by centrifugation twice at 6000 g then aliquoted and stored at -80°C until used for antibody neutralization analysis.

*Antibody neutralization assay using VSVΔG-RFP SARS-CoV-2:* All sera were heat-inactivated for 30 min at 55°C prior to use in neutralization assay. Vero E6 cells stably expressing TMPRSS2 were seeded in 100 μl at 2.5x10^4^ cells/well in a 96 well collagen coated plate. The next day, 2-fold serially diluted serum samples were mixed with VSVΔG-RFP SARS-CoV-2 pseudotype virus (100-300 focus forming units/well) and incubated for 1hr at 37°C. Also included in this mixture to neutralize any potential VSV-G carryover virus was 1E9F9, a mouse anti-VSV Indiana G, at a concentration of 600 ng/ml (Absolute Antibody, Ab01402-2.0). The serum-virus mixture was then used to replace the media on VeroE6 TMPRSS2 cells. 22 hours post infection, the cells were washed and fixed with 4% paraformaldehyde before visualization on an S6 FluoroSpot Analyzer (CTL, Shaker Heights OH). Individual infected foci were enumerated and the values compared to control wells without antibody. The focus reduction neutralization titer 50% (FRNT_50_) was measured as the greatest serum dilution at which focus count was reduced by at least 50% relative to control cells that were infected with pseudotype virus in the absence of human serum. FRNT_50_ titers for each sample were measured in at least two technical replicates and were reported for each sample as the geometric mean of the technical replicates.

### Detection of SARS-CoV-2-Specific Memory B Cells

Antigen-specific B cells were detected using biotinylated proteins in combination with different streptavidin (SA)-fluorophore conjugates. Biotinylated proteins were multimerized with fluorescently labeled SA for 1 hour at 4C. Full-length spike protein (R&D Systems) was mixed with SA-BV421 (Biolegend) at a 10:1 mass ratio (e.g., 200ng spike with 20ng SA; ~4:1 molar ratio). Spike RBD (R&D Systems) was mixed with SA-APC (Biolegend) at a 2:1 mass ratio (e.g., 25ng RBD with 12.5ng SA; ~4:1 molar ratio). Biotinylated influenza HA pools were mixed with SA-PE (Biolegend) at a 6.25:1 mass ratio (e.g., 100ng HA pool with 16ng SA; ~6:1 molar ratio). Individual influenza HA antigens corresponding with the 2019 trivalent vaccine (A/Brisbane/02/2018/H1N1, B/Colorado/06/2017; Immune Technology) were biotinylated using an EZ-Link Micro NHS-PEG4 Biotinylation Kit (Thermo Fisher) according to the manufacturer’s instructions. Excess biotin was subsequently removed using Zebra Spin Desalting Columns 7K MWCO (Thermo Fisher) and protein was quantified with a Pierce BCA Assay (Thermo Fisher). SA-BV711 (BD Bioscience) was used as a decoy probe without biotinylated protein to gate out cells that non-specifically bind streptavidin. All experimental steps were performed in a 50/50 mixture of PBS + 2% FBS and Brilliant Buffer (BD Bioscience). Antigen probes for spike, RBD, and HA were prepared individually and mixed together after multimerization with 5uM free D-biotin (Avidity LLC) to minimize potential cross-reactivity between probes. For staining, 5x10^6^ cryopreserved PBMC samples were prepared in a 96-well U-bottom plate. Cells were first stained with Fc block (Biolegend, 1:200) and Ghost 510 Viability Dye (Tonbo Biosciences, 1:600) for 15 min at 4C. Cells were then washed and stained with 50uL antigen probe master mix containing 200ng spike-BV421, 25ng RBD-APC, 100ng HA-PE, and 20ng SA-BV711 decoy for 1 hour at 4C. Following incubation with antigen probe, cells were washed again and stained with anti-CD3 (BD Bioscience, 1:200), anti-CD19 (Biolegend, 1:100), anti-CD20 (BD Bioscience, 1:500), anti-CD27 (BD Bioscience, 1:200), anti-CD38 (BD Bioscience, 1:200), anti-CD71 (BD Bioscience, 1:50), anti-IgD (BD Bioscience, 1:50), anti-IgM (Biolegend, 1:200), and anti-IgG (Biolegend, 1:400). After surface stain, cells were washed and fixed in 1% PFA overnight at 4C. For sorting, cells were stained with spike and HA probes followed by Fc block and Ghost 510 Viability Dye as described above. Cells were then stained for surface markers with anti-CD4 (Invitrogen, 1:333.3), anti-CD8 (Biolegend, 1:66.7), anti-CD14 (Biolegend, 1:200), anti-CD19 (BD Bioscience, 1:100), anti-CD27 (Biolegend, 1:66.7), and anti-CD38 (1:200). After surface stain, cells were washed and resuspended in PBS + 2% FBS for acquisition. All antibodies and recombinant proteins are listed in **table S1** and **table S2**.

### Flow Cytometry and Cell Sorting

Samples were acquired on a BD Symphony A5 instrument. Standardized SPHERO rainbow beads (Spherotech) were used to track and adjust photomultiplier tubes over time. UltraComp eBeads (Thermo Fisher) were used for compensation. Up to 5x10^6^ cells were acquired per sample. Data were analyzed using FlowJo v10 (BD Bioscience). Antigen-specific gates were set based on healthy donors stained without antigen probes (similar to an FMO control) and were kept the same for all experimental runs. All timepoints for individual subjects were run in the same experiment to minimize batch effects. The full gating strategy is shown in **figure S2**. Cell sorting was performed on a BD FACSAria II instrument in low pressure mode, using a 70 μm nozzle. SARS-CoV-2-specific memory B cells were similarly identified as live, CD14^-^, CD19^+^, CD27^+^ CD38^lo/int^, HA^-^ Spike^+^. Cells were sorted into 1.5 DNA LoBind Eppendorf tubes containing 300 μl of cell lysis buffer (Qiagen) and stored at room temperature until nucleic acid extraction.

### B Cell Receptor (BCR) Sequencing

DNA was extracted from sorted cells using Gentra Puregene Cell kit (Qiagen, Cat.# 158767). Immunoglobulin heavy-chain family-specific PCRs were performed on genomic DNA samples using primers in FR1 and JH as described previously (*46*, *47*). Two biological replicates were run on all samples. Sequencing was performed in the Human Immunology Core Facility at the University of Pennsylvania using the Illumina 2 × 300-bp paired-end kit (Illumina MiSeq Reagent Kit v3, 600-cycle, Illumina MS-102-3003).

### B Cell Receptor (BCR) Sequence Analysis

Raw reads from the Illumina MiSeq were quality controlled with pRESTO v0.6.0 (*48*) as described in (*49*). Sequences passing the quality control procedure were imported into IgBLAST v1.17.0 (*50*) for gene identification and alignment. The primer binding region (IMGT nucleotide positions 1-80) was replaced with *N*s and sequences beginning after IMGT position 90 were removed to avoid incorrect V-gene calls and skewed SHM analysis. The remaining sequences were imported into ImmuneDB v0.29.10 (*51*) for clonal inference, lineage construction, and downstream analyses. Sequences sharing the same VH-gene, JH-gene, CDR3 length, and 85% amino-acid homology in the CDR3 were aggregated into clones. After sequences were collapsed into clones, non-productive sequences and clones with 1 copy number sequences were excluded from all downstream analysis.

Lineages were constructed within ImmuneDB as described in (*51*). Within each lineage, sequences with fewer than ten copies across all samples in a donor were excluded to reduce the effect of sequencing error and improve fidelity. The resulting lineage structures were visualized with ete3 (*52*). Each node represents a unique sequence and the size of each node is proportional to the total copy number of the sequence. Nodes are depicted as pie-charts where each wedge indicates the proportion of copies at each timepoint and inferred nodes are shown in black. The number next to each node represents the number of nucleotide mutations as compared to the preceding vertical node.

### Data Visualization and Statistics

All antibody and memory B cell data were analyzed using custom scripts in R Studio. B cell receptor sequencing data were analyzed as discussed above. Data were visualized using ggplot2 in R Studio. Boxplots represent median with interquartile range. Line plots represent means with a 95% confidence interval. For heatmaps, data were scaled by variable (z-score normalization) and cells with z > 3.5 were assigned a maximum value of 3.5. For principal component analysis, data were also scaled by variable (z-score normalization). Statistical tests are indicated in the corresponding figure legends. All tests were performed two-sided with a nominal significance threshold of p < 0.05. In all cases of multiple comparisons, adjustment was performed using Holm correction. For comparisons between timepoints, unpaired tests were used due to missing data/samples for some participants. * indicates p < 0.05, ** indicates p < 0.01, *** indicates p < 0.001, **** indicates p < 0.0001. ns indicates no significant difference. Blue and red values indicate statistical comparisons within naïve or recovered groups. Black values indicate statistical comparisons between naïve or recovered groups. Source code are available upon request from the authors. All raw data are provided in **table S4**.

## SUPPLEMENTARY MATERIALS

immunology.sciencemag.org/cgi/content/full/6/58/eabi6950/DC1 Supplemental Figure 1. Cohort design and summary statistics. Supplemental Figure 2. Identification of antigen-specific B cells. Supplemental Figure 3. Clonal evolution of spike-binding memory B cell lineages that were present prior to vaccination in a recovered individual. Supplemental Figure 4. B cell populations in sex and age subgroups. Supplemental Figure 5. Relationships between antibody and memory B cell responses following mRNA vaccination. Supplemental Table 1. Flow cytometry panel used for antigen-specific B cell assays. Supplemental Table 2. Recombinant proteins used for antigen-specific B cell assays. Supplemental Table 3. B cell receptor sequencing metadata. Supplemental Table 4: Raw data file.
